# Draft Genome Sequences of Two Methicillin-Resistant Clinical *Staphylococcus aureus* Isolates

**DOI:** 10.1128/genomeA.01396-15

**Published:** 2016-02-11

**Authors:** Saeed Khan, Kidon Sung, Saira Iram, Mohamed Nawaz, Joshua Xu, Bernard Marasa

**Affiliations:** a National Center for Toxicological Research (NCTR), Division of Microbiology, U.S. Food and Drug Administration, Jefferson, Arkansas, USA; b Department of Microbiology, Quaid-i-Azam University, Islamabad, Pakistan; c Division of Bioinformatics and Biostatistics, NCTR, U.S. Food and Drug Administration, Jefferson, Arkansas, USA; d Center for Drug Evaluation and Research (CDER), Division of Microbiological Assessment, Office of Process and Facilities, U.S. Food and Drug Administration, Silver Spring, Maryland, USA

## Abstract

Here, we report the draft genome sequences of two methicillin-resistant *Staphylococcus aureus* (MRSA) clinical isolates, hospital-associated perirectal isolate 32S (ST 239) from a colitis tracheostomy patient and community-associated MRSA isolate 42S (ST 772) from a hepatic-splenomegaly patient in Rawalpindi, Pakistan.

## GENOME ANNOUNCEMENT

*Staphylococcus aureus* is one of the leading human pathogens that causes a broad variety of infections, ranging from minor skin infections to life-threatening conditions, such as endocarditis, pneumonia, toxic shock syndrome, and bloodstream infections (1–3). Molecular characterization of the virulence and antimicrobial resistance gene profiles and the draft genome sequences of methicillin-resistant *S. aureus* (MRSA) isolates will enhance the understanding of their evolution and aid in rapid diagnosis, detection, and development of better therapeutic intervention strategies. The isolates described here were highly resistant to oxacillin (MIC, 512 mg/L) and ciprofloxacin (MIC, >256 mg/L), and also exhibited resistance to ampicillin, tetracycline, penicillin, erythromycin, gentamicin, and kanamycin. Sequence analysis of the quinolone resistance determinant region of the target gene *gyrA* indicated a double point mutation (S84L and G106D) in 32S and a single point mutation in 42S (S84L). The *grlA* gene exhibited E84G mutation in 32S and S80Y in 42S. RT-qPCR analysis of the efflux pump genes, *norA, norB, norC,* and *mdeA*, indicated an increased efflux pump expression in these isolates (4).

Both of the isolates were positive for staphylococcal cassette chromosome type SCC*mec* III and harbored various toxin genes (*eta*, *sea*, *seb*, *sec*, *hla*, *hlb*, *hld*, *hlg*, *pvl*, *lukS, lukF, lukM, lukE-D*, *sed*, *see*, *seg*, *seh*, and *tst*), adhesin genes (*ebpS*, *bbp*, *cna*, *clfA*, *clfB*, *fnbA*, *fnbB*, *map-eap*, and *spa*), and virulence genes (*chp*, *ica*, *cfb*, and *v8*). Multilocus sequence typing (MLST) as described in Enright et al. (5) indicated an ST 239 profile for 32S and ST 772 for 42S. The sequence analysis of the 3′ coding region X of the *spa* gene with the online *spa*-typing program (http://spatyper.fortinbras.us) revealed the *spa* type t030 for 32S and t5414 for 42S.

The genomic DNAs from *S. aureus* were extracted using a Master Pure Gram-positive DNA purification kit (Epicentre Biotechnologies). The library was prepared using a TruSeq DNA library preparation kit (Illumina) and the TruSeq paired-end cluster kit was used for the DNA preparation and cluster generation. The Illumina HiSeq 2500 system was used for sequencing. CLC Genomics Workbench 6.5.1 (Qiagen) was used for the *de novo* assembly of high-quality 100 bp paired-end reads and further genome annotation was performed using an annotation method GeneMarkS+ in the NCBI Prokaryotic Genome Annotation Pipeline (6).

The draft genome sequence of *S. aureus* 32S is 2,943,897 bp in length which is distributed in 118 contigs (average coverage, 20.0×) with 3,082 genes, 2,912 CDs, 110 pseudo genes, 37 frameshifted genes, 3 rRNAs (5S,16S, 23S), and 56 tRNAs. The draft genome sequence of *S. aureus* 42S has 32.7% G+C content, is 2,766,469 bp in length which is distributed in 86 contigs (average coverage, 50×) with 2,816 genes, 35 frame-shifted genes, 63 pseudo genes, 2,692 CDs, 6 rRNAs (5S,16S, 23S), and 55 tRNAs.

### Nucleotide sequence accession numbers.

This whole-genome shotgun project was deposited at DDBJ/EMBL/GenBank under the accession numbers JTJX00000000 and JTFJ00000000 for 32S and 42S, respectively. The versions described in this paper are versions JTJX02000000 and JTFJ01000000.

## References

[1] Klevens RM, Morrison MA, Nadle J, Petit S, Gershman K, Ray S, Harrison LH, Lynfield R, Dumyati G, Townes JM, Craig AS, Zell ER, Fosheim GE, McDougal LK, Carey RB, Fridkin SK, Active Bacterial Core Surveillance (ABCs) MRSA Investigators 2007 Invasive methicillin-resistant *Staphylococcus aureus* infections in the United States. JAMA 298:1763–1771. doi:10.1001/jama.298.15.1763. 17940231

[2] Lowy FD 1998 *Staphylococcus aureus* infections. N Engl J Med 339:520–532. doi:10.1056/NEJM199808203390806. 9709046

[3] Gordon RJ, Lowy FD 2008 Pathogenesis of methicillin-resistant *Staphylococcus aureus* infection. Clin Infect Dis 46:S350–S359. doi:10.1086/533591. 18462090 PMC2474459

[4] Marasa BS, Iram S, Sung K, Kweon OG, Cerniglia CE, Khan S 2015 Molecular characterization of fluoroquinolone resistance of methicillin-resistant clinical *Staphylococcus aureus* isolates from Rawalpindi, Pakistan. Med Res Arch 2:1–14.

[5] Enright MC, Day NP, Davies CE, Peacock SJ, Spratt BG 2000 Multilocus sequence typing for characterization of methicillin-resistant and methicillin-susceptible clones of *Staphylococcus aureus*. J Clin Microbiol 38:1008–1015. 10698988 10.1128/jcm.38.3.1008-1015.2000 PMC86325

[6] Angiuoli SV, Gussman A, Klimke W, Cochrane G, Field D, Garrity G, Kodira CD, Kyrpides N, Madupu R, Markowitz V, Tatusova T, Thomson N, White O 2008 Toward an online repository of standard operating procedures (SOPs) for (meta) genomic annotation. Omics 12:137–141. doi:10.1089/omi.2008.0017. 18416670 PMC3196215

